# Comprehensive DNA methylation analysis of hepatitis B virus genome in infected liver tissues

**DOI:** 10.1038/srep10478

**Published:** 2015-05-22

**Authors:** Surbhi Jain, Ting-Tsung Chang, Sitong Chen, Batbold Boldbaatar, Adam Clemens, Selena Y. Lin, Ran Yan, Chi-Tan Hu, Haitao Guo, Timothy M. Block, Wei Song, Ying-Hsiu Su

**Affiliations:** 1 JBS Science, Inc., Doylestown, Pennsylvania; 2 Department of Internal Medicine, National Cheng Kung University Medical College and Hospital, Tainan, Taiwan, Republic of China; 3 Department of Microbiology and Immunology, Drexel University College of Medicine, Philadelphia, Pennsylvania; 4 Department of Medicine, Buddhist Tzu Chi General Hospital and Tzu Chi University, Hualien, Taiwan, Republic of China

## Abstract

Hepatitis B virus (HBV) is a hepatotropic virus causing hepatitis, cirrhosis and hepatocellular carcinoma (HCC). The methylation status of the HBV DNA in its different forms can potentially provide insight into the pathogenesis of HBV-related liver diseases, including HCC, however this is unclear. The goal of this study is to obtain comprehensive DNA methylation profiles of the three putative CpG islands in the HBV DNA in infected livers, with respect to liver disease progression. The extent of methylation in these CpG islands was first assessed using bisulfite PCR sequencing with a small set of tissue samples, followed by analysis using both quantitative bisulfite-specific PCR and quantitative methylation-specific PCR assays in a larger sample size (n = 116). The level of HBV CpG island 3 methylation significantly correlated with hepatocarcinogenesis. We also obtained, for the first time, evidence of rare, non-CpG methylation in CpG island 2 of the HBV genome in infected liver. Comparing methylation of the HBV genome to three known HCC-associated host genes, *APC*, *GSTP1*, and *RASSF1A*, we did not identify a significant correlation between these two groups.

Despite the availability of a preventive vaccine, chronic Hepatitis B virus (HBV) infection remains a significant global health issue, affecting more than 350 million people worldwide. If left unmonitored or untreated, approximately one-third of these chronically infected individuals will develop end-stage liver disease, such as cirrhosis or HBV-related HCC (HBV-HCC)^1^,^2^,^3^,^4^, which is associated with more than 600,000 deaths annually^5^,^6^.

The role of HBV infection in the pathogenesis of HBV-HCC is believed to be multifactorial. During carcinogenesis, genetic and epigenetic modifications of the genome occur not only in the host, but also in the virus. The mutations in the basal core promoter (i.e. A1762T/G1764A), precore (i.e. G1899A), or deletions in the preS2 region have been associated with HCC^7^,^8^,^9^,^10^,^11^,^12^,^13^,^14^. Although previous studies have identified epigenetic modifications of HBV DNA, including methylation, in both cell cultures and HCC tissue, the association between the methylation of HBV DNA and the progression of liver disease to HCC has not yet been fully explored^15^,^16^,^17^. This could be partially due to the complexity of HBV DNA in diseased tissue^18^,^19^,^20^.

This complexity can be linked to the presence of ten genotypes of HBV, and cases have been reported of co-infection both by multiple genotypes and recombination between genotypes^18^. Due to using an error-prone reverse transcriptase in viral DNA replication, HBV has a high mutation rate, resulting in quasispecies distribution in an infected individual^19^,^21^. Furthermore, there are multiple replicative intermediates of HBV DNA in the viral life cycle. In patients, HBV circulates in blood as virion DNA and exists in hepatocytes both in nuclear DNA forms (episomal cccDNA, deproteinized double-stranded DNA including relaxed circular DNA and linear DNA, and integrated linear DNA) and in cytoplasmic core DNA forms^21^.

The HBV genome has 2-3 typical CpG islands depending on the genotype^17^,^22^. Interestingly, these CpG islands are located at strategic locations in the regulatory elements of the HBV genome. For example, CpG island 1 (CG1) is located in the first exon start site for the S (surface antigen) gene, and CpG islands 2 (CG2) and 3 (CG3) cover the enhancer II and the promoter of pregenomic RNA and the first exon start site of the polymerase gene, respectively^17^,^23^. Although the virion DNA was found to be mostly unmethylated in both tissue culture and patient serum^16^,^17^, DNA methylation of the intranuclear HBV genome has been associated with repression of gene transcription in cultures^24^,^25^,^26^. Despite the difficulty of dissecting HBV DNA in diseased tissues, higher levels of methylation of CG1 were found in HCC tissues compared to hepatitis and cirrhosis tissues^15^,^16^,^17^. Vivekanandan *et al.* showed the detection of methylation of the CG2 in total DNA isolated from HCC tissues^17^. In addition, methylation of the CG2 of cccDNA was found to be significantly higher in HBeAg-negative patients than in HBeAg-positive patients^17^,^25^. To our knowledge, only 2 studies have studied the methylation of CG3 in HBV-HCC tissue, but neither of them have reported an association between CG3 methylation and HCC^15^,^16^.

This study was set out to obtain comprehensive HBV DNA methylation profiling of 73 CpG sites in three CpG islands and then to correlate these profiles to liver disease progression. To conquer the diversity in HBV DNA sequences in patient samples, we first performed genotyping through DNA sequencing, and we then designed and performed bisulfite (BS) specific sequencing accordingly for all 3 CpG islands. Lastly, we developed quantitative methylation specific PCR (qMSP) assays for each of the 3 CpG islands to assess methylation in a larger sample size. We found that only the methylation of CG3 was significantly higher in HCC as compared to hepatitis and cirrhosis tissues. To our knowledge, this is the first study demonstrating the significant association of HBV CG3 methylation with HCC. Strikingly, we discovered, for the first time, evidence of non-CpG methylation of the HBV genome derived from the infected liver tissues. Additionally, we found no significant correlation between the HBV DNA methylation status and DNA methylation of three HCC-associated host genes, *adenomatous polyposis coli* (*APC*), *glutathione S-transferas*e π 1 (*GSTP1*), and *RASSF1A*, in tissue DNA samples.

## Results

### HBV genotyping and methylation profile analysis of the HBV DNA derived from different HCC cell lines by BS-PCR sequencing

**Figure 1:**
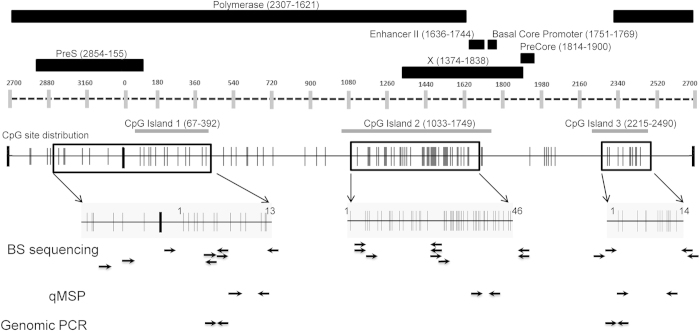
Diagram of the HBV genome indicating the location of the three CpG islands, CpG site distribution, and the primers for PCR assays used in this study. **** Black rectangles represent the HBV regions; polymerase, enhancer II, basal core promoter, precore, pre-S and X gene. These regions correspond to the dashed line representing the HBV genome with vertical gray bars indicating nucleotide location. The three CpG islands are indicated by horizontal grey bars below the dashed line. The CpG site distribution in the HBV genome (solid black line) is depicted by vertical grey lines each representing an individual CpG site. The boxes with black borders on the CpG distribution map indicate the regions targeted by the bisulfite sequencing PCR and are enlarged below for better resolution of the CpG sites. The numbering of the CpG sites in the enlarged regions corresponds to that used in Fig. 2. The arrows below this enlarged bisulfite sequencing region indicate primer locations for the specified PCR assays. Detailed primer information is available in Supplementary Table S3.

To obtain the comprehensive methylation profiles for each CpG site of all three known CpG islands in the HBV genomes from both cell cultures and infected tissues, we chose to perform bisulfite PCR (BS-PCR) sequencing on a small set of tissue samples (8 hepatitis, 6 cirrhosis, and 12 HCC) based on the quantity of DNA available to us. The clinicopathological information is summarized in Supplementary Table S1. In order to design the suitable BS-specific primers, we began by performing the genotyping of the HBV DNA in each tissue sample, as described in Materials and Methods. We then designed BS primers (total of 24 sets) covering three CpG islands in various genotypes of the HBV genome, to amplify the BS-treated HBV DNA from cell cultures and tissue samples (Fig. 1, Supplementary Table S3).

**Figure 2:**
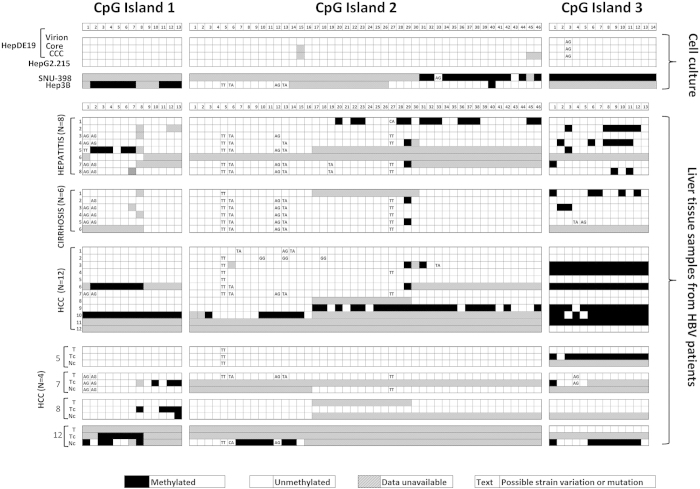
Methylation profile of the HBV genome by bisulfite sequencing. **** Each box indicates one CpG site (filled, methylated; open, unmethylated; hatch, data unavailable; text, possible sequence variations at CpG site). The top panel shows the results from cell cultures –HepDE19 cell line derived virion, core, cccDNA and total DNA from HepG2.215, SNU-398 and Hep3B aligned to genotype D. The next three panels show the results from clinical samples (hepatitis, cirrhosis and HCC) aligned to genotype C. The bottom panel (HCC) illustrates results from matched total tumor tissue DNA (T), cccDNA from both tumor (T_C_) and adjacent non-HCC tissue (N_C_) obtained from the livers of the 4 HCC subjects (#5, 7, 8 and 12) as indicated in the HCC panel above, which is also aligned to genotype C.

To analyze the BS-PCR sequencing data, we numbered the CpG sites for each CpG island (Figs. 1,2). Genotype D was used as the reference genome for HBV DNA isolated from HepDE19, Hep3B, HepG2.2.15, and SNU398 cells, as this is the genotype in all four cultures. We used genotype C for the HBV DNA sequences obtained from patient samples since this was the most prevalent strain identified in the HCC tissues examined. Among four major types of HBV DNA, virion, core, and cccDNA were derived from HepDE19 cells, and the integrated DNA was derived from Hep3B and SNU398 cells. Total DNA from HepG2.215 cells, which contains all forms of HBV DNA, was also included. BS-PCR sequencing data for 73 CpG sites are summarized in Fig. 2.

Consistent with a previous study^17^, we did not detect methylation in virions. No detectable methylation was identified either in cytoplasmic core, the cccDNA/extra-chromosomal nuclear DNA isolated from HepDE19 cells, or the total DNA from the HepG2.2.15 cell culture. For the integrated DNA, when the DNA was derived from SNU-398 cells, which contains the transcriptionally repressed HBV genome^27^, DNA methylation was detected in all the HBV DNA amplified by BS-PCR primers. When the integrated HBV DNA from Hep3B was analyzed, which is known to express the S antigen, preS and X transcripts^28^, the HBV DNA was mostly unmethylated except for the CG1, which was mostly methylated. As shown in Fig. 2, there are regions marked as “data unavailable” such as the CG1 and the 5’ end of the CG2 of the HBV genome in SNU-398 cells, and the middle of the CG2 and CG3 of the HBV genome in Hep3B cells. These were the regions that BS-PCR reactions either failed to generate specific PCR products for DNA sequencing, or the sequencing data were unreadable despite multiple attempts. Many attempts using multiple sets of primers for both untreated and BS-treated DNA were unable to amplify detectable PCR products, perhaps due to the possibility that DNA sequences in these areas might be either deleted during HBV integration, or altered to the extent that none of the tested primers had sufficient homology to prime the PCR reaction. As compared to the reference genome, few nucleotide variants were noted in Hep3B and SNU-398 cells.

### Methylation profile of the HBV DNA derived from HBV-infected diseased liver tissue by bisulfite-PCR sequencing

We next performed BS-PCR sequencing analysis on the diseased tissues. As aforementioned, we developed a total of 24 sets of primers based on genotyping data, 10 sets for the CG1, 12 sets for the CG2, and 2 sets for the CG3. Although we were able to generate specific PCR products from most of the HBV infected tissue DNA to obtain BS sequencing data for most of the CpG sites, there were still regions in the genomes that failed to be amplified, regardless of multiple attempts. Thus, the methylation status was unavailable for those regions, as indicated in Fig. 2. Nevertheless, the information obtained is sufficient for statistical analysis of the methylation of each CpG island comparing HCC tissues to hepatitis and cirrhosis.

To compare DNA methylation in different disease groups, we calculated the percentage of CpG sites that were found to contain detectable levels of methylation, which were analyzed for each disease group within each CpG island (Table 1). A low level of methylation was detected in all three CpG islands in the hepatitis samples (6.6%, 6%, and 17.5% in CG1, CG2 and CG3, respectively), whereas, the HBV DNA detected in cirrhosis tissue was mostly unmethylated in CG1 (0%) and CG2 (0.8%), and a low level of methylation was found in CG3 (10.8%). Interestingly, in the HCC samples, although a low percentage of methylation was detected in CG1 and CG2 (16.1% and 8%, respectively), 52.5% of the CpG sites in CG3 were found to be methylated. Furthermore, every CpG site of CG3, except for three, were found to be methylated in 6 of the 12 HCC samples that had methylation. Comparing these three CpG islands within HCC samples, the CG3 is the most methylated (p < 0.001, comparing CG3 to CG1 and CG3 to CG2, by Fisher’s two-tailed exact test). When comparing the extent of methylation of CG3 among three disease groups, HCC was significantly higher than that of hepatitis and cirrhosis (*p* < 0.0001, Fisher’s two-tailed exact test, Table 1). Similarly, HCC also had significantly higher levels of methylation in CG1 and CG2, as compared to hepatitis and cirrhosis (CG1, p = 0.0007; CG2, p = 0.0046, Fisher’s two-tailed exact test, Table 1). There were few nucleotide variants with respect to the reference genome (Genbank # NC_003977.1), as noted in Fig. 2, which were mostly in CG2 and the first 2 CpG sites of CG1.

### cccDNA methylation profile in HCC tissue

DNA methylation analysis shown in Fig. 2 and Table 1 is for total HBV DNA detected in the infected tissue. cccDNA is critical for HBV DNA replication and its methylation is known to repress gene transcription. However, currently no study has compared the methylation of cccDNA to matching total HBV DNA obtained from HCC tissues. Hence, it was of interest to investigate the methylation of cccDNA, or extra-chromosomal HBV DNA, in HCC tissue, and then to compare the methylation pattern of cccDNA between HCC tumor tissue and its matched, adjacent non-tumor tissue to that of total HBV DNA. We isolated extra-chromosomal cccDNA from 4 HCC tissues (Tc, #5, 7, 8 and 12) and the corresponding adjacent non-tumor tissues (Nc), based on the availability of a large amount of tissue specimen available for cccDNA isolation, as described in Materials and Methods. For the purpose of comparison, we aligned the total HBV DNA (t) and cccDNA from tumor (Tc) and cccDNA from matched adjacent non-tumor (Nc) together for each of the four patients (Fig. 2). Interestingly, although many sequencing data are not available for statistical comparison, due to failures in generating specific PCR products from BS-PCR primers, regardless of many attempts with many primer sets, the methylation pattern of HBV cccDNA is not identical to that of total HBV DNA from the same tissue. For example, in patient 5, significant CG3 methylation was detected in cccDNA at a higher level than was detected in total DNA. In addition, the methylation of cccDNA was different between tumor and adjacent non-tumor tissues.

### Discovery of Non-CpG methylation in the HBV genome in patients with HBV related liver diseases

**Figure 3:**
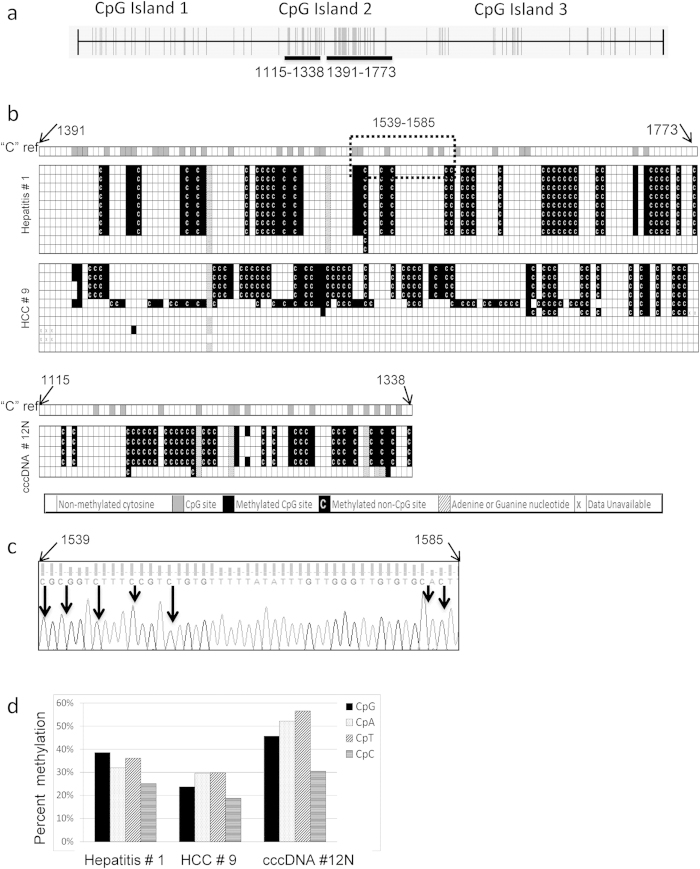
Evidence of non-CpG methylation in the HBV genome from infected livers. **** (**a**) CpG distribution map of the HBV genome depicting each individual CpG site by a vertical line. Horizontal black bars illustrate the regions that were identified as containing non-CpG methylation (nt. positions 1115-1338 and 1391-1773). (**b**) The locations of non-CpG methylation as referred to the cytosine map (“C” ref). For the boxes corresponding to “C” ref each box indicates a cytosine site in which an open box indicates a non-CpG cytosine (CpA, CpT, or CpC) and a grey box indicates a CpG cytosine (CpG). The samples are numbered as in Fig. 2. The bisulfite sequencing of each clone is presented below the “C” ref. A filled box indicates a methylated CpG site. A filled box with a white C indicates a methylated non-CpG cytosine. An open box indicates unmethylated cytosine. A hatched box indicates a change in the sequence to adenine or guanine. Boxes with an “x” indicate invalid sequencing data. (**c**) Example of the sequencing chromatogram (nt. 1539-1585) of the hepatitis #1, clone 1, as indicated by the dotted box in Fig. 3b. Arrows indicate methylated cytosine dinucleotides. (**d**) The percentage of methylation at both CpG and non-CpG sites (CpA, CpT and CpC) as observed in the three liver samples obtained from hepatitis # 1, HCC # 9 and cccDNA # 12N.

An intriguing feature we noted while performing BS-PCR sequencing analysis was that we found non-CpG methylations in CG2 of the HBV genome in three of the tissue DNA samples that we analyzed. These samples were hepatitis #1, HCC #9, and cccDNA from #12N. The regions identified to contain non-CpG methylation are indicated in Fig. 3a. These non-CpG methylations were not due to incomplete bisulfite conversion, as all bisulfite sequencing had a conversion rate of at least 97%. This was determined by the percent of converted cytosines in non-CpG cytosine sites located in other regions of the HBV DNA, as the detailed method described in our previous work^29^. In order to confirm these non-CpG methylations, we cloned PCR products from BS-PCR reactions and isolated DNA from an individual clone for DNA sequencing. Five to ten clones for each sample were analyzed (Fig. 3b). In addition to CpG methylation in each sample, approximately 31% of the non-CpG cytosines were methylated in the hepatitis #1, 27% in HCC #9, and 48% in the cccDNA isolated from adjacent non-tumor tissue #12. A representative chromatogram of one clone from each sample is shown in Supplementary Fig. S1, where every non-CpG methylation is shaded. As an example, methylation was identified in CpG, CpT, CpC, and CpA in the nt. 1539 to 1585 from hepatitis #1 (Fig. 3c). Next, we investigated whether any particular non-CpG cytosine was preferred for methylation by calculating the percentage of cytosine methylation in each of these three DNA samples. The non-CpG methylation does not appear to be biased towards any particular non-CpG dinucleotide (Fig. 3d).

### Quantitative measurement of the extent of DNA methylation of the HBV genome in various liver disease tissues

To evaluate whether the extent of DNA methylation was associated with the progression of liver diseases, we developed quantitative MSP (qMSP) assays for each CpG island to measure the DNA methylation in a larger sample size. As some of the HBV infected tissues may not contain any detectable HBV DNA, each DNA sample was first subjected to the quantitative HBV DNA PCR assays for CG1 and CG3, as described in the Materials and Methods. Only samples positive for at least one HBV DNA assay were subjected to methylation analysis. The clinicopathological characteristics of the study subjects (74 HCC, 29 hepatitis and 13 cirrhosis) are listed in Supplementary Table S2.

**Figure 4:**
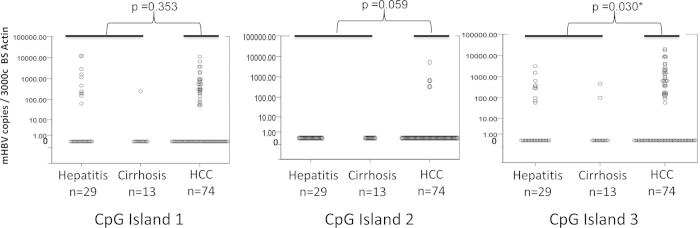
Quantitative analysis of HBV DNA methylation in infected diseased livers. **** Scatter plots of the quantity of methylated HBV DNA detected by qMSP assays for each CpG island. Each data point represents one sample and is the average of two duplicate assays per input of 3000 copies of BS-actin DNA. p values indicate the statistical comparison between HCC and non-HCC (hepatitis and cirrhosis) samples for each CpG island by Mann-Whitney U test.

Three qMSP assays were performed to quantify the level of methylation for each of the three CpG islands. As shown in Fig. 4, there is no statistically significant difference (p > 0.05, Mann Whitney U-test) between the methylation levels of HCC and non-HCC (hepatitis and cirrhosis) tissues in CG1 and CG2. In contrast, methylation of CG3 in HCC tissues (mean = 1250.5, standard deviation = 3504) was significantly higher than in non-HCC tissue (hepatitis and cirrhosis, mean = 174.6, standard deviation = 534) tissues (p < 0.05, Mann Whitney U-test). Of the 74 HBV-HCC cases, 9 are HCV positive, 45 are HCV negative, and the HCV status of the remaining 20 is unknown. HCV infection is also known to cause aberrant methylation and can be a potential confounding factor. Hence, we compared the methylation levels of CG3 in HBV-positive, HCV-negative HCC (n = 45) to HBV-infected hepatitis and cirrhosis samples. The results confirmed that methylation of CG3 in this subset of HCC tissues was still significantly higher than in hepatitis and cirrhosis tissues (*p* < = 0.05, Mann Whitney U-test). We also observed no significant differences in the levels of CG3 methylation in the HBV-HCC samples that were categorized by their HCV status (*p* *=* 0.695, Kruskal Wallis test). Interestingly, we observed higher levels of methylation in CG1 and CG3 of hepatitis samples as compared to cirrhosis samples, although only methylation of CG1 was significantly higher in hepatitis samples as compared to cirrhosis samples (p = 0.05, Mann Whitney U test). No methylation was observed in either hepatitis or cirrhosis samples in CG2.

### Correlation between HBV and host genome methylation in HBV-HCC

**Figure 5:**
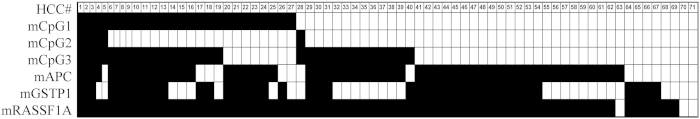
Comparison of HBV and host gene methylation in HBV-HCC. **** Each column represents one sample of the 71 HBV-HCC. Each row depicts the overall methylation status of either the viral CpG islands (mCpG1/2/3) or host gene promoter regions (mAPC/mGSTP1/mRASSF1A). Filled rectangles indicate methylation detected and open rectangles indicate methylation below the level of detection.

It has been shown that HBV infection upregulates DNA methyltransferase activity, which leads to simultaneous methylation of HBV DNA and host CpG islands in cell culture experiments^30^. DNA methylation of many tumor suppressor genes has been associated with carcinogenesis. Among these known tumor-associated, aberrant-methylation events, methylation of the *APC, GSTP-1*, and *RASSF1A* genes were found to be associated with HCC^29^,^31^,^32^,^33^. It is therefore of interest to investigate whether the HBV DNA methylation correlates with these three HCC-associated host gene methylation events. BS-treated HCC DNA was subjected to previously developed quantitative MSP assays for these three genes (Fig. 5), as described in Materials and Methods. The Spearman’s *rho* test was used to determine the correlation co-efficiency (Table 2). When comparing methylation of genes within the host genome, there is a significant correlation (*p* < 0.01) between either of the two host genes, and within the HBV genome in HBV-related HCC, there is also a significant correlation (*p* < 0.01) between CG1 and CG3 of the HBV genome in HCC. The only significant correlation between HBV DNA and host genes was found between methylation of CG3 and *RASSF1A.*

## Discussion

Our comprehensive analysis of DNA methylation of three CpG islands of the HBV genome, first done by the bisulfite-PCR sequencing, followed by qMSP assays in both cell cultures and infected livers, demonstrated that among three CpG islands, CG3 is the most methylated in HCC tissues. This is the first report, to our knowledge, that demonstrates that the CG3 methylation in HCC tissue (n = 74) is higher to a degree of statistical significance (*p* < 0.05) than that of hepatitis (n = 29) and cirrhosis (n = 13) tissues. Strikingly, we discovered non-CpG methylation in CG2 of the HBV genome in infected liver. Upon comparison of the extent of DNA methylation between HBV DNA and three HCC-associated host genes, *APC*, *GSTP-1*, and *RASSF1A,* we did not detect a significant correlation.

The result obtained from BS-PCR sequencing and confirmed by quantitative MSP assays in a larger sample size study can be summarized below.

Firstly, CG2, which overlaps with the X gene and the basal core promoter region and acts to regulate the pregenomic RNA transcription, is minimally methylated across the entire spectrum of HBV-related liver diseases. This minimal CG2 methylation favors a notion that the HBx gene is transcriptionally active and also allows for pre-genomic RNA transcription to proceed throughout the progression of liver disease to HCC. This is consistent with one previous study that minimal methylation of CG2 was detected in chronic-infected liver tissues^17^.

Secondly, our study demonstrated that, by BS sequencing, only methylation of CG1 and CG3 was significantly associated with HCC as compared to hepatitis and cirrhosis (p < 0.001). While methylation of CG1 in HCC is consistent with previous work^15^,^16^,^17^, methylation of CG3 in HCC is in contrast with two previous studies that did not report any association between methylation of CG3 and HCC^15^,^16^. This could be due to their limited HCC sample size (n = 5^15^ and n = 8^16^) and/ or due to different study populations. Interestingly, when comparing the methylation between HCC and hepatitis with cirrhosis by qMSP assays, we only observed a statistically significant increase in the methylation of CG3. These discrepancies could be due to the differences in sensitivity of the two methods. Nevertheless, it is clear that methylation of the CG2 was not significant regardless of the disease stages.

Thirdly, there is a statistically significantly correlation of methylation among three HCC-associated host genes. Similarly, methylation of CG1 and CG3 also has a statistically significant correlation. However, there is a lack of significant, positive correlation between HBV methylation and host genome methylation as a group. It will be interesting to study if this lack of correlation between host and HBV genome methylation is consistent in a more comprehensive host gene panel.

Strikingly, here we discovered the evidence of non-CpG methylation in the HBV DNA obtained from liver tissues including a cccDNA form of the virus. In the mammalian genome, cytosine methylation is restricted largely to the CpG dinucleotide, although non-CpG methylations have been reported in cultured pluripotent stem cells. These include embryonic stem cells and induced pluripotent stem cells^34^ in the mouse germ line^35^,^36^,^37^, as well as in the adult mouse cortex^35^ and human brain^38^,^39^. Interestingly, the non-CpG methylation was found only in the CG2 of 3 tissue samples among the 26 studied in BS-PCR sequencing, and the three samples were derived from different diseases: one from hepatitis, one was cccDNA from adjacent non-HCC cirrhotic tissue, and one from HCC tissue. Thus, the non-CpG methylation event is not a frequent event. Since three non-CpG methylation cases were in three different liver disease tissues, it is difficult to relate the non-CpG methylation identified to its role in liver pathogenesis, however, non-CpG methylation was not detected in any of the HBV DNA derived from all four cell cultures (HepDE19, HepG2.215, Hep3B and SNU398).

Besides our finding here, the HPV genome detected in cervical cancer was the only another human virus found to contain non-CpG cytosine methylation, except for the viral vector transfection system^40^,^41^. Similar to CpG methylation, non-CpG methylation was also associated with transcription repression *in vitro*. In addition, non-CpG methylation is believed to be non-inherent, thus it was suggested to be an indication of *de novo* methylation^42^,^43^. When non-CpG methylation was detected, the frequency of cytosine methylation, in a given DNA was suggested to be CpG > CpA > CpT > CpC^44^. Given the small sample size, we did not see any preference for any dinucleotide. Further study is needed to understand the potential significance of the non-CpG methylation of the HBV genome in pathogenesis of infected liver.

Our sequencing data from the various forms of HBV DNA, which is consistent with previous studies, suggest that the HBV DNA is mostly unmethylated during its productive phases. This unmethylated form of HBV DNA is also found in most patients with hepatitis and cirrhosis. By comparing two HCC cell lines with the integrated HBV genomes, specifically the SNU398, which has low or no expression of HBV genes^27^ and the Hep3B line, which expresses the S antigen, preS, and X transcripts of the HBV genome^28^, the HBV DNA in transcriptionally repressed SNU398 cells was found to be extensively methylated, whereas the HBV DNA in Hep3B cells was only partly methylated in the CG1. These data, together with previous studies^25^,^30^ indicate that DNA methylation is associated with silencing of the HBV genome.

The integrated HBV genome is also known to have deletions and insertions due to the non-homologous, end-joining mediated integration^45^. The reduced complexity of the DNA sequences after bisulfite conversion (very few C’s and many T’s), together with high-homology regions of the human genome, especially in CG2, and the presence of several genotypes/quasispecies, makes it difficult to have a set of BS-specific primers to successfully amplify the HBV genome of interest in infected tissues. In this study, a total of 24 sets of primers were designed for the CG1 and CG2 after genotyping of HBV DNA in each of the 26 tissue DNA samples. There are still regions that BS-PCR reactions failed to generate specific PCR products for sequencing, thus resulting in “data unavailable” for analysis (Fig. 2). The CG3 is known to be conserved among HBV strains, so only two sets of primers were needed for the region to generate DNA methylation profiles for most of the tissue DNA samples. This could be one of the reasons why only a handful of studies^15^,^16^,^17^ that have looked into the methylation of the HBV genome in infected livers, only a few^16^,^17^ were able to report the methylation data from HCC tissue. This might also be the reason why approximately 60% of the HBV genome was found to be deleted in most of HCC tissues studied^15^.

Although there are regions marked as “data unavailable” for methylation analysis by BS-PCR sequencing, our data provide a comprehensive analysis of the methylation profile of the HBV genome in both tissue cultures and diseased tissue. The result obtained from BS-PCR sequencing is confirmed by quantitative MSP assays in a larger sample size study. Similarly, not enough sequencing data are available for the proper comparison of HBV cccDNA with total HBV DNA in infected tissues. Due to the limitation of available data and the small sample size (n = 4), a statistical analysis was not applicable for the comparison of whether the methylation of cccDNA found in HCC tissue is at a higher level than that of cccDNA detected in adjacent non-tumor tissue. Nevertheless, these data, although not surprisingly, indicate for the first time that methylation of cccDNA could be different from methylation of total HBV DNA in infected liver tissue, and the methylation levels could be different between tumor and adjacent non-tumor tissues as well.

In conclusion, a significant increase in DNA methylation of CpG island 3 in HCC tissues, as compared to hepatitis and cirrhosis liver tissues, has been demonstrated, implicating HBV methylation in HBV-HCC pathogenesis. Based on our observations, we speculate that the regulation of HBV genome methylation does not significantly correlate with host genome methylation. This suggests that the methylation status of CpG island 3 could be used as a biomarker in combination with HCC-associated host gene methylation for HBV-HCC.

## Materials and Methods

### Cell lines and study subjects

HepDE19 and HepG2.2.15 cells were maintained as previously described^46^. Hep3B and HepG2 cells were originally obtained from ATCC. The SNU-398 cell line was provided by Immunotope, Inc (Doylestown, PA). All patient samples (HCC, n = 74; cirrhosis, n = 13, hepatitis, n = 29) were acquired under institutional review board (IRB) approvals from the National Cheng-Kung University Hospital, Tainan, Taiwan, and the Buddhist Tzu Chi Medical Center in Hualien, Taiwan. The diagnosis of liver diseases was performed by pathologists. The clinicopathological characteristics of patient samples are provided in Supplementary Table S1 for BS-PCR sequencing study and in Supplementary Table S2 for qMSP PCR assay. Tissue samples for cccDNA and total DNA for 4 HCC samples were previously described^29^.

### DNA isolation and Bisulfite (BS) treatment

Cell culture derived virion, core, and cccDNA were prepared from HepDE19, as previously described^46^. Briefly, virion DNA was obtained from the culture medium, capsid-associated core DNA was extracted from the cytoplasmic fraction, and the cccDNA was isolated from the nuclear fraction with subsequent plasmid-safe DNase digestion (Epicentre, Madison, WI). The total culture and tissue DNA were isolated using the DNeasy Blood and Tissue kit (Qiagen, Valencia, CA). BS treatment was performed using EZ DNA Methylation-Lightning™ Kit (Zymo Research, Irvine, CA).

### Bisulfite specific polymerase chain reaction (BS-PCR) sequencing and cloning

BS-PCR reactions were performed using the primers listed in Supplementary Table S3, using Qiagen HotStarTaq Plus Master Mix Kit (Qiagen). PCR products were purified using the QIAquick PCR purification kits. Purified PCR product was either sent for sequencing at the NAPCore Facility at the Children’s Hospital of Philadelphia (Philadelphia, PA) or cloned using the Strataclone kit (Agilent, Santa Clara, CA), transformed, screened for the inserted DNA and sequenced. Sequencing results were analyzed using ClustalW software (available at http://www.ch.embnet.org/) and Finch TV version 1.4.0 (Geospiza, Inc., Seattle, WA).

### Genotyping

Genotyping was performed by DNA sequencing analysis. The nucleotide (nt.) position 57-273 of the HBV genome was amplified using previously described PCR primers in the conserved regions^20^ followed by Sanger sequencing. The DNA sequences were analyzed by BLAST analysis against the NCBI nucleotide collection (http://blast.ncbi.nlm.nih.gov/) for subtyping of HBV genotype^18^.

### Quantification of HBV DNA and DNA methylation

Quantitative BS-specific PCR (qBS-PCR) and qMSP assays for all three CpG islands were developed based on the conserved DNA sequences among four genotypes, A (AY934774), B (AB602818.1), C (NC_003977.1) and D (U95551.1). The sequences of the primers, probes, and assay conditions are listed in Supplementary Table S3. To prepare for methylated HBV DNA, the total DNA from HepG2.2.15 was methylated by the SssI methylase (NEB, Ipswich, MA). The amount of HBV DNA per HepG2.2.15 host DNA was determined to be 200 copies through real-time PCR quantification of globin gene^47^ and HBV DNA quantification kit (JBS Science, Inc. Doylestown, PA). The copy number of methylated HBV DNA after BS treatment was determined by the quantity of BS-Actin quantification^29^ times 200 copies per HepG2.2.15 cell. Reconstituted standards were prepared using methylated HepG2.2.15 BS DNA and HepG2 BS DNA. The amount of HBV DNA in each sample was quantified using HBV DNA quantification kit (JBS Science, Inc. Doylestown, PA) with primers for the HBV pol/S and HBV core regions.

### Quantification of the methylation of *APC, GSTP1* and *RASSF1A* genes

The extent of DNA methylation of three HCC-related host genes, *APC, GSTP1*, and *RASSF1A*, was determined by previously developed qMSP assays for *APC*^29^, *GSTP1*^31^ and *RASSF1A*^48^ . The input DNA for each assay was 300 copies of BS-treated DNA, as determined by a previously developed BS-actin qPCR assay^29^. The amount of methylated DNA was calculated from the average of duplicated reactions.

### Statistical Analysis

Statistical Analysis was performed using Graph Pad software (La Jolla, CA). Methylation analysis was evaluated by a two-tailed Fisher’s exact test. Mann Whitney U-test, Kruskal Wallis test and Spearman’s correlation test were all performed using the SPSS Statistics 20 software (IBM, Armonk, NY).

## Additional Information

**How to cite this article**: Jain, S. *et al.* Comprehensive DNA methylation analysis of hepatitis B virus genome in infected liver tissues. *Sci. Rep.* **5**, 10478; doi: 10.1038/srep10478 (2015).

## Supplementary Material

Supporting Information

## Figures and Tables

**Table 1:** HBV DNA methylation in various HBV-infected livers by BS-PCR sequencing.

**Percent of methylated CpG sites**€	**CpG island**		
	**1**	**2**	**3**
Hepatitis	6.6% (5/76)	6.0% (15/250)	17.5% (16/91)
Cirrhosis	0% (0/60)	0.8% (2/236)	10.8% (7/65)
HCC	16.1% (20/124)	8.0% (32/398)	52.5% (75/143)
p value# HCC vs. (Hepatitis and Cirrhosis)	0.0007	0.0046	<0.0001

^€^The percent of methylated CpG sites was calculated as the number of methylated CpG sites detected / total CpG sites with valid BS sequencing data (methylated + unmethylated) shown in Fig. 2.

^#^Fisher’s two-tailed exact test.

**Table 2:** Correlation of DNA methylation of the HBV genome with three known HCC associated host genes in HCC tissue.

**Genes**	**Statistic**	**mCpG1 (n=74)**	**mCpG3 (n=74)**	**mAPC (n=71)**	**mGSTP1 (n=71)**
mCpG3 (n =74)	Spearman’s *rho*	.471			
	p value	<0.001			
mAPC (n =71)	Spearman’s *rho*	0.098	0.094		
	p value	0.418	0.435		
mGSTP1 (n =71)	Spearman’s *rho*	0.233	-0.008	.324	
	p value	0.051	0.947	0.006	
mRASSF1A (n =71)	Spearman’s *rho*	0.132	.269	.510	.513
	p value	0.271	0.023	<0.001	<0.001
