# Supplementary material for: Comprehensive DNA methylation analysis of hepatitis B virus genome in infected liver tissues
Source: Sci Rep. 2015 May 22;5:10478. doi: 10.1038/srep10478 (PMC4650678; doi:10.1038/srep10478)
Supplement: Supporting Information [file srep10478-s1.pdf]

Comprehensive DNA methylation analysis of hepatitis B virus genome in infected liver tissues

Surbhi Jain, Ting-Tsung Chang, Sitong Chen, Batbold Boldbaatar, Adam Clemens, Selena Y. Lin, Ran Yan, Chi-Tan Hu, Haitao Guo, Timothy M. Block, Wei Song, Ying-Hsiu Su

Supplementary data

Supplementary Figure Legend

Supplementary Figure S1. Illustration of non-CpG methylation as representative chromatograms from three HBV-infected liver tissues obtained by BS-PCR cloning and sequencing. The sample numbers (as in Fig. 2) and the regions examined are indicated on the left. Shaded regions indicate methylated non-CpG sites.

Supplemental Figure S1

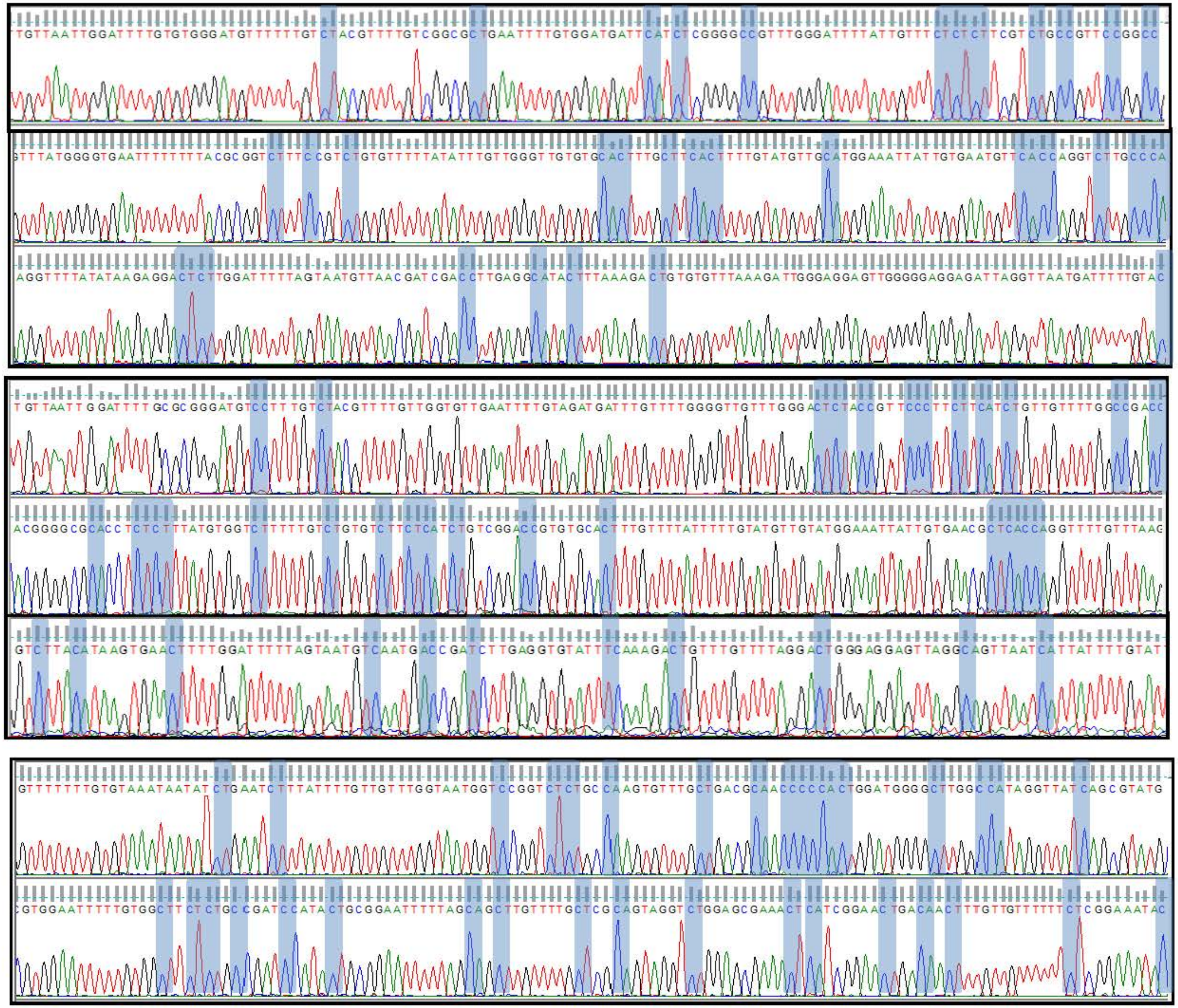

Hepatitis # 1
1391-1773

HCC # 9
1391-1773

cccDNA # 12N
1115-1338

Supplementary Table S1. Clinicopathological characterization of the tissues analyzed by BS-PCR DNA sequencing

Characteristic	Hepatitis (n = 8)	Cirrhosis (n = 6)	HCC (n = 12)
Mean age (years) ± SD	49.9 ± 16.2	58.1 ± 11.2	49.9 ± 12.5
Male/female (n)	5/3	4/2	9/3
HBV only/ HBV+HCV/ HCV status not known (n)	3/5/0	6/0/5	12/0/10
Stage 1/2/3/4/unknown (n)	—	—	0/8/0/0/4
Grade 1/2/3/unknown (n)	—	—	0/2/0/10
AFP levels ≤ 20/ > 20 ng/ml/unknown (n)	—	—	0/2/10

SD. Standard deviation; HBV, hepatitis B virus; HCV, hepatitis C virus.

Supplementary Table S2. Clinicopathological characterization of the tissues analyzed by quantitative MSP assays

	**Hepatitis (n = 29)**	**Cirrhosis (n =13)**	**HCC (n =74)**
Mean age ± SD	45.2 ±14	48.9 ±15	57.1 ± 11
Male/female (n)	22/7	9/4	54/20
HBV only/ HBV+HCV/ HCV status not known (n)	21/8/3	9/4/5	65/9/20
Stage 1/2/3/4/unknown (n)	—	—	25/33/12/2/2
Grade 1/2/3/unknown (n)	—	—	10/46/16/2
Mean size of tumor ± SD	—	—	4.9 ± 3.5 cm
AFP levels ≤20 ng/ml / >20 ng/ml / unknown	—	—	39/332/3

SD. Standard deviation; HBV, hepatitis B virus; HCV, hepatitis C virus.

Supplementary Table S3. Primers, probes and PCR conditions .

Assay	Location	Forward primer	Nucleotide position	Reverse primer	Nucleotide position	PCR conditions	Taqman Probe/ Detection format	Nucleotide position
BS-sequencing	CpG Island 1	ATTTTTTTGTTGGTGGTTTTAG	51-72	AATCCAAAAAAACCAATAAAAA	431-452	95°C 5 min, (95°C 30s, 50 °C 30s, 72°C 30s) x 40 cycles	Agarose Gel and Sanger sequencing	NA
		ATTTTTTTGTTGGTGGTTTTAG	51-72	TAAAAAATTAAAAAAAATCCACCAC	256-280			
		ATTTTTTTGTTGGTGGTTTTAG	51-72	AAATTTTTATCAACAAAAAAAACC	207-230			
		GTGGTGGATTTTTTTTAATTTTTTAGG	256-282	TAAAAAATTAAAAAAAATCCACCAC	256-280			
		GTGGTGGATTTTTTTTAATTTTTTAGG	256-282	AATCCAAAAAAACCAATAAAAA	431-452			
		ATTTTTAGGTTATGTAGTGGAA	3194-3215	AAATTTTTATCAACAAAAAAAACC	207-230			
		ATTTTTAGGTTATGTAGTGGAA	3194-3215	TAAAAAATTAAAAAAAATCCACCAC	256-280			
		GTGGAGTTTTTAGGTTTAGGGTATAT	3075-3100	AAATTTTTATCAACAAAAAAAACC	207-230			
		GTGGAGTTTTTAGGTTTAGGGTATAT	3075-3700	TAAAAAATTAAAAAAAATCCACCAC	256-280			
		GGTTTTTTTTGTTGATAAGAATT	207-230	AATCCAAAAAAACCAATAAAAA	431-452			
	CpG Island 2	AGGTTTTTTTGTGTAAATAATAT	1112-1134	CAAAATCCAATTAACAACA	1389-1407	95°C 5 min, (95°C 30s, 50 °C 30s, 72°C 30s) x 40 cycles		
		AGGTTTTTTTGTGTAAATAATAT	1112-1134	AAACCAATTTATACCTACAACCTCC	1776-1800			
		AGGTTTTTTTGTGTAAATAATAT	1112-1134	TAAAAACCCAAACGACCC	1467-1484			
		AGGTTTTTTTGTGTAAATAATAT	1112-1134	ACACCAATTTATACCTACAACCTCC	1776-1800			
		AGGTTTTTTTAAGTAAATAGTAT	1112-1134	TAAAACCCCAAACGACCC	1467-1484			
		AGGTTTTTTTAAGTAAATAGTAT	1112-1134	AAACCAATTTATACCTACAACCTCC	1776-1800			
		TAGGTTTTTGTTAAGTGTTTGTTG	1166-1181	TAAAACCCCAAACGACCC	1467-1484			
		TAGGTTTTTGTTAAGTGTTTGTTG	1166-1181	ACACCAATTTATACCTACAACCTCC	1776-1800			
		TGGTTGTTAGGTTGTGTTGTTAAT	1375-1398	AAACCAATTTATACCTACAACCTCC	1776-1800	95°C 5 min, (95°C 30s, 54 °C 30s, 72°C 30s) x 40 cycles		
		TGGTTGTTAGGTTGTGTTGTTAAT	1375-1398	ACACCAATTTATACCTACAACCTCC	1776-1800			
		GTTGTGTTGTTAATTGGATTTT	1385-1406	AAACCAATTTATACCTACAACCTCC	1776-1800			
		GTTGTGTTGTTAATTGGATTTT	1385-1406	ACACCAATTTATACCTACAACCTCC	1776-1800			
	CpG Island 3	AGGTAAGTTATTTTTTGTTGGG	1966-1987	CCAATAAAATTTCCCACCTTATAAT	2463-2488	95°C 5 min, (95°C 30s, 51 °C 30s, 72°C 30s) x 40 cycles		
		TTGTTTTTGAGTATTTGGTGTTTTTT	2241-2264	CCACCTTATAAATCCAAAAAAT	2454-2466	95°C 5 min, (95°C 30s, 54 °C 30s, 72°C 30s) x 40 cycles		
Genotyping	CpG Island 1	CTGCTGGTGGCTCCAGTT	57-74	TTGAGAGAAGTCCACCACGAG	253-273	95°C 5 min, (95°C 30s, 58 °C 30s, 72°C 30s) x 40 cycles		
MSP	CpG Island 1	ACGTGTTTTGGTTAAAATTCGTAGTTTTTA	292-322	AATATAATAAAACGCCGCAAACACATC	376-402	95°C 5 min, (95°C 10s, 55°C 30s, 72°C 10s) x 50 cycles	[6FAM] GTTTTTTAATTTGTTTTGGTTATCGTTGGATG [BHQ1]	348-379
	CpG Island 2	TGTCGTTTCGGTCGATTAC	1502-1520	CACGATCCGACAAATAAAAA	1560-1579	95°C 5 min, (95°C 10s, 52°C 30s, 72°C 10s) x 45 cycles, Melting curve, 40°C 30s	Sybr Green	NA
	CpG Island 3	GTGTGGATTCGTATTTTTTTC	2270-2290	GACGATTAAAACCTTCGTCT	2393-2412	95°C 10 min, (95°C 10s, 53°C 30s, 72°C 10s) x 45 cycles	[6FAM]AACCTACCTCGTCGTCTAACAACAAT[BHQ1]	2339-2364
